# Molecular Differentiation of *Mycobacterium tuberculosis* Strains without IS*6110* Insertions

**DOI:** 10.3201/eid0811.020291

**Published:** 2002-11

**Authors:** Kerry H. Lok, William H. Benjamin, Michael E. Kimerling, Virginia Pruitt, Monica Lathan, Jafar Razeq, Nancy Hooper, Wendy Cronin, Nancy E. Dunlap

**Affiliations:** * University of Alabama at Birmingham, School of Medicine, Birmingham, Alabama, USA; † Alabama Department of Public Health, Montgomery, Alabama, USA; ‡ Maryland Department of Health and Mental Hygiene, Baltimore, Maryland, USA

**Keywords:** IS*6110*, zero band, *Mycobacterium tuberculosis*, molecular epidemiology

## Abstract

By using standard restriction fragment length polymorphism, 6 zero-copy IS*6110* *Mycobacterium tuberculosis* isolates were identified from 1,180 Maryland isolates as part of the National Tuberculosis Genotyping Surveillance Network Project. By using various genotyping methods, we demonstrated that this zero band cluster can be differentiated into six genotypes.

IS*6110* restriction fragment length polymorphism (RFLP) has been used to investigate *Mycobacterium tuberculosis* transmission within populations since the early 1990s (1–4). However, some strains do not have any IS*6110* insertions and thus are zero-band strains (5) and are considered a single IS*6110* cluster by exclusion (5,6). These zero-band strains are rare in the United States and usually are found in persons from Southeast Asia. Recently, the spacer oligonucleotide typing (spoligotyping) technique was used to divide some zero-copy IS*6110* strains into subclusters that are associated with distinct geographic origins. As part of the National Tuberculosis Genotyping Surveillance Network, six zero-copy IS*6110* strains were identified in persons from Maryland during a 5-year period. We conducted further molecular characterization of these strains to ascertain if they were closely related.

Six zero-copy IS*6110* isolates were identified from 1996 to 2000. These isolates, negative for IS*6110* by RFLP analysis, were from 1,180 Maryland cases. The isolates were collected as part of the genotyping network. Of the six patients, three were from Vietnam (two shared a common surname), and the others came from India, Iraq, and Liberia. None were linked epidemiologically to one another.

We identified all isolates as *M. tuberculosis* complex with BACTEC NAP test (BACTEC 460, BD Diagnostic Systems, Sparks, MD) and AccuProbe (Gen-Probe, San Diego, CA) before they were sent to the Alabama Regional Genotyping Laboratory. The isolates were fingerprinted at least twice by IS*6110* RFLP to rule out technical error in the RFLP procedure. After error was ruled out, all zero-copy strains were then tested with three secondary typing methods.

*M. tuberculosis* isolates were cultured on Lowenstein-Jensen or 7H11 Middlebrook plates for at least 4 weeks before DNA extraction. Chromosomal DNA was extracted from the isolates with chloroform-isoamyl alcohol, and RFLP was performed according to international standards (7). For the zero-copy IS*6110* strains, membranes with negative lanes were subjected to at least twice the normal exposure time to rule out the possibility of missing a faint band.

The use of spoligotyping was based on the presence or absence of 43 variable spacers in the direct repeat (DR) region of *M. tuberculosis*. Spoligotyping membranes were purchased from Isogen Bioscience BV (Isogen, Bilthoven, the Netherlands). We followed the manufacturer’s recommendations for hybridizing polymerase chain reaction (PCR) products, as described by Groenen and colleagues (8). The numbering of the spacer regions was done as reported previously (8). Excel (Microsoft Corp., Redmond, WA) was used to analyze the spoligotyping results. For national database reporting, we converted the spoligotyping image into an octal-digital format based on the protocol set by colleagues and the genotyping network (9).

After *Alu*I digestion, the DNA was transferred to a nylon membrane for polymorphic guanine cytosine-rich repetitive sequence (PGRS) Southern blotting. Southern-blotting protocol was followed at the genotyping network except for the use of the plasmid pTBN12 as the probe. The results were compared visually (10).

The variable number of tandem repeats (VNTR) typing method was employed, as previously described, to further investigate these strains (11). The sizes of the fragments were determined by using a DNA ladder and amplicons from strain H37Rv (11).

Four different spoligotype patterns were obtained for the six isolates (Table). Three isolates had the same spoligotype patterns (designated Centers for Disease Control and Prevention [CDC] spoligotype 258): isolates 1–3 with an absence of spacers 19–41. The other three patterns were distinct with deletion of spacers as follows: isolate 4 (deletions at 4–11, 13–36, and 40); isolate 5 (deletions at 12–43); and isolate 6 (deletions at 33–34).

**Table:** Zero-copy IS*6110* *Mycobacterium tuberculosis* strains from previously reported studies, other studies from the National Tuberculosis Genotyping Surveillance Network, and strains from current study^a^

No./case	Spoligotype octal description	CDC designation	Country of origin	Yr reported	Source of data
1	N/A	N/A	India	1993	(10)
3	N/A	N/A	Hong Kong	1995	(5)
5	N/A	N/A	India	1995	(5)
3	577767000000011	N/A	Asia	1997	(12)
1	777777777760771	0002	Curacao	1999	(13)
1	477776501013071	N/A	India	1999	(13)
1	777701002001731	N/A	the Netherlands	1999	(13)
1	N/A	N/A	United States	Unpublished	Arkansas
1	777777777760700	0202	China	Unpublished	California
8	777777000000011	0258	Vietnam	Unpublished	California
1	777603000000011	1682	Vietnam	Unpublished	California
1	777777600007771	1128	North Korea	Unpublished	California
1	777777000000011	0258	Vietnam	Unpublished	Michigan
1	777603002000011	0870	Vietnam	Unpublished	Texas
1	N/A	N/A	Vietnam	Unpublished	Texas
1	000000000003771	0034	N/A	2000	(17)
2	777777000000011	0258	Vietnam	2001	(18)
1	777603000000011	1682	Vietnam	2001	(18)
1	741777000000011	n/a	Vietnam	2001	(18)
2	777647000000011	n/a	United States	2001	(18)
3	777777000000011	0258	Vietnam	2002	Study isolates 1–3
1	700100000000731	0968	Iraq	2002	Study isolates 4
1	577600000000000	0742	India	2002	Study isolates 5
1	777777777763771	0169	Liberia	2002	Study isolates 6
43					

^a^CDC; Centers for Disease Control and Prevention.

PGRS also yielded four patterns and divided the strains into groups that were identical to those found with spoligotyping (Figure 1). Three isolates (lanes 1–3) had the same banding pattern, and one isolate (lane 6) had a similar pattern that differed only by the presence of a double rather than single band at 2,760 bp. The two remaining isolates had distinct patterns (lanes 4 and 5). The lack of variability in the PGRS patterns suggests that these strains may be related. The genomes of these strains may be more stable than strains with IS*6110*.

**Figure 1:**
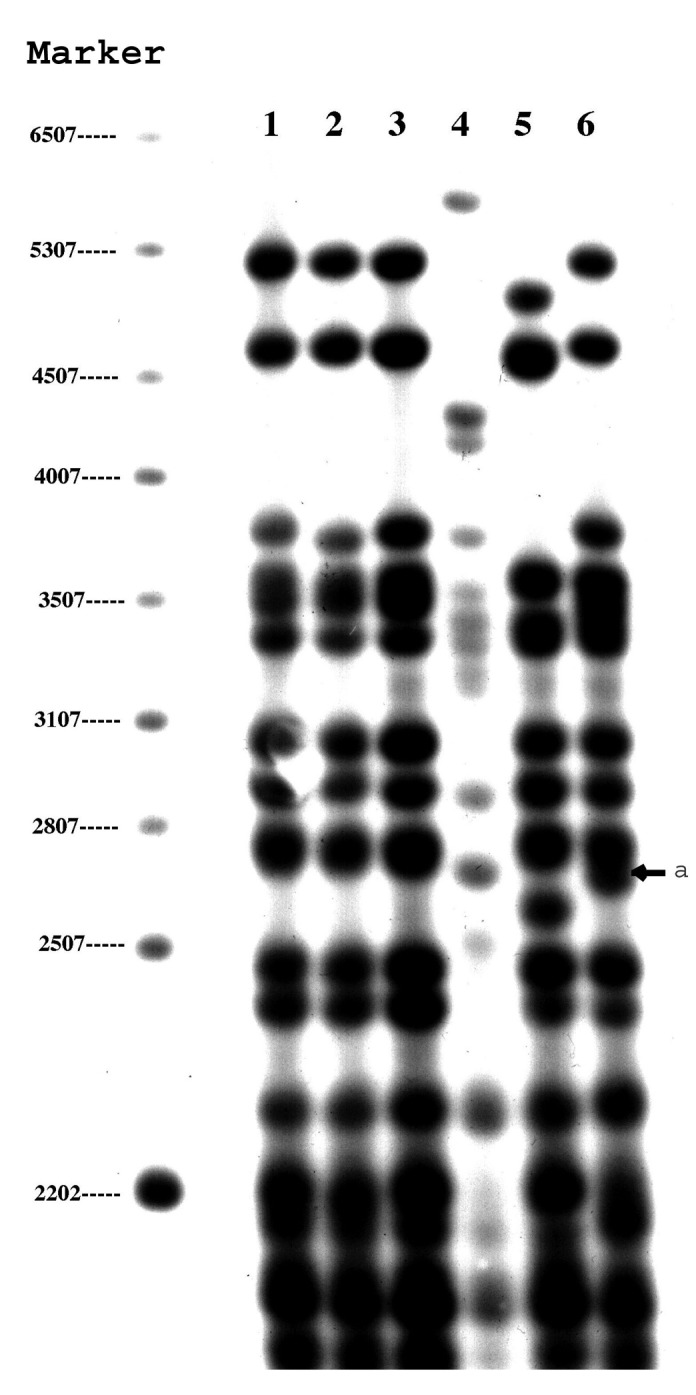
Polymorphic guanine cytosine-rich repetitive sequence restriction fragment length polymorphism results of six zero-copy IS*6110* strains. Lanes 1–6 represent the six cases reported in this study. The arrow indicates an additional band at 2,760 bp in isolate 6 compared to lanes 1–3.

We used VNTR to genotype the three samples (lanes 1–3) (Figure 1) that had identical spoligotype and PGRS results. The profile of these three isolates, which were obtained from three Vietnamese patients, were differentiated only by loci exact tandem repeats (ETR)-A and ETR-D (Figure 2). The remaining three isolates (not shown) had multiple differences.

**Figure 2:**
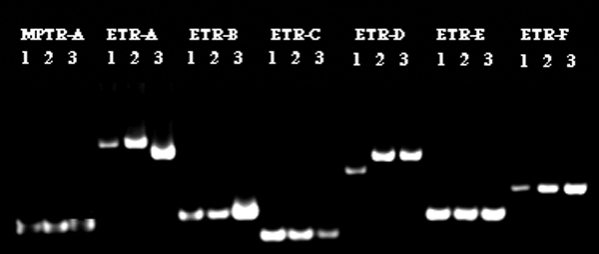
The variable number of tandem repeats results of three zero-copy IS*6110* isolates with identical spoligotyping and polymorphic guanine cytosine-rich repetitive patterns. Seven VNTR loci are listed across the top. Second line represents the patient number of the isolates for each locus.

The first zero-copy IS*6110* *M. tuberculosis* strain was reported by van Soolingen and colleagues in 1993 (10). Subsequently, 22 cases have been reported (5,6,12–14). Within the genotyping network, another 21 cases were documented between 1996 and 2000. California reported 11 cases, Maryland 6 (this study), Texas 2, Michigan 1, and Arkansas 1. Zero-copy isolates were not found in New Jersey and Massachusetts. These isolates represent only 21 (0.18%) of 11,923 fingerprinted from seven surveillance sites. Therefore, zero-copy IS*6110* strains are extremely rare in the United States. Goguet de la Salmoniere and colleagues reported three zero-copy IS*6110* from 106 cases (2.8%) in three French hospitals during a 1-year study (12). This rate is 10 times higher compared with the genotyping network findings in the United States. The combined data of spoligotype profiles and the patients’ countries of origin suggest that most isolates originated in Asia (15). Without additional epidemiologic data, this hypothesis cannot be corroborated.

Using the secondary molecular genotyping techniques, we showed that all six isolates were different. We found that all strains but one had deletions in the DR region that included DR 24 (a common and perhaps original insertion site for IS*6110)*. Only the strain from Liberia has spacer 24 in the DR region. This strain represented an exception in the zero-copy IS*6110* strains. However, the absence of spacer 24 is not an absolute indication of a zero-copy IS*6110* strain. All Beijing strain families have a deletion that includes spacers 1–34, but they also have multiple IS*6110* insertions (15 to 21) and some in the remaining DR region (16).

Among the six zero-copy IS*6110* strains, the three Vietnamese isolates had the same spoligotype and PGRS. By using VNTR genotyping we were able to differentiate these strains; two VNTR loci differentiated them. The importance of this finding is unknown.

In comparing the six zero-copy IS*6110* cases reported in this study with more recent data from the genotyping network, we identified 43 zero-copy IS*6110* strains (Table). Most of these strains (35 [81%] of 43) originated from southern Asia. Fourteen of 32 spoligotyped strains are similar and have the same CDC designation. Additionally, eight of the strains are similar to CDC spoligotype 258, distinguished by the same absence of spacers 19–41. Most strains spoligotyped (29 [91%] of 32) have a deletion of the spacer 24 region. The other three isolates did have spacer 24, which cannot be explained without sequencing the DR region.

By using multiple genotyping methods, we confirmed the identification of zero-copy *M. tuberculosis* isolates. We further demonstrated that the six unrelated cases were caused by different *M. tuberculosis* strains. However, the close similarity of the Vietnamese genotypes implies an important geographic association. An optimal algorithm for evaluating zero band isolates is yet to be determined and should be based on evolving secondary methods.

## References

[1] Mazurek GH, Cave MD, Eisenach KD, Wallace RJJ, Bates JH, Crawford JT. Chromosomal DNA fingerprint patterns produced with IS*6110* as strain-specific markers for epidemiologic study of tuberculosis. J Clin Microbiol. 1991;29:2030–3. 1663520 10.1128/jcm.29.9.2030-2033.1991 PMC270253

[2] Dunlap NE. The use of RFLP as a tool for tuberculosis control: utility or futility? Int J Tuberc Lung Dis. 2000;4:S134–8. 11144543

[3] Small PM, Hopewell PC, Singh SP, Paz A, Parsonnet J, Ruston DC, The epidemiology of tuberculosis in San Francisco. A population-based study using conventional and molecular methods. Stability of DNA fingerprint pattern produced with IS*6110* in strains of *Mycobacterium tuberculosis.* N J Med. 1994;330:1703–9. 10.1056/NEJM199406163302402 7910661

[4] Jasmer R, Hahn J, Small P, Daley C, Behr M, Moss A, A molecular epidemiologic analysis of tuberculosis trends in San Francisco, 1991–1997. Ann Intern Med. 1999;130:971–8. 10383367 10.7326/0003-4819-130-12-199906150-00004

[5] Das S, Paramasivan CN, Lowrie DB, Prabhakar R, Narayanan PR. IS*6110* restriction fragment length polymorphism typing of clinical isolates of *Mycobacterium tuberculosis* from patients with pulmonary tuberculosis in Madras, south India. Tuber Lung Dis. 1995;76:550–4. 10.1016/0962-8479(95)90533-2 8593378

[6] Das S, Chan SL, Allen BW, Mitchison DA, Lowrie DB. Application of DNA fingerprinting with IS986 to sequential mycobacterial isolates obtained from pulmonary tuberculosis patients in Hong Kong before, during and after short-course chemotherapy. Tuber Lung Dis. 1993;74:47–51. 10.1016/0962-8479(93)90068-9 8098637

[7] van Embden JD, Cave MD, Crawford JT, Dale JW, Eisenach KD, Gicquel B, Strain identification of *Mycobacterium tuberculosis* by DNA fingerprinting: recommendations for a standardized methodology. J Clin Microbiol. 1993;31:406–9. 8381814 10.1128/jcm.31.2.406-409.1993 PMC262774

[8] Groenen PM, Bunschoten AE, van Soolingen D, van Embden JD. Nature of DNA polymorphism in the direct repeat cluster of *Mycobacterium tuberculosis*; application for strain differentiation by a novel typing method. Mol Microbiol. 1993;10:1057–65. 10.1111/j.1365-2958.1993.tb00976.x 7934856

[9] Dale JW, Brittain D, Cataldi AA, Cousins D, Crawford JT, Driscoll J, Spacer oligonucleotide typing of bacteria of the *Mycobacterium tuberculosis* complex: recommendations for standardised nomenclature. Int J Tuberc Lung Dis. 2001;5:216–9. 11326819

[10] van Soolingen D, de Haas P, Hermans P, Groenen P, van Embden JD. Comparison of various repetitive DNA elements as genetic markers for strain differentiation and epidemiology of *Mycobacterium tuberculosis.* J Clin Microbiol. 1993;31:1987–95. 7690367 10.1128/jcm.31.8.1987-1995.1993 PMC265684

[11] Frothingham R, Meeker-O'Connell WA. Genetic diversity in the *Mycobacterium tuberculosis* complex based on variable numbers of tandem DNA repeats. Microbiology. 1998;144:1189–96. 10.1099/00221287-144-5-1189 9611793

[12] Goguet de la Salmoniere YO, Li HM, Torrea G, Bunschoten A, van Embden J, Gicquel B. Evaluation of spoligotyping in a study of the transmission of *Mycobacterium tuberculosis.* J Clin Microbiol. 1997;35:2210–4. 9276389 10.1128/jcm.35.9.2210-2214.1997 PMC229941

[13] Kremer K, van Soolingen D, Frothingham R, Haas WH, Hermans PW, Martin C, Comparison of methods based on different molecular epidemiological markers for typing of *Mycobacterium tuberculosis* complex strains: interlaboratory study of discriminatory power and reproducibility. J Clin Microbiol. 1999;37:2607–18. 10405410 10.1128/jcm.37.8.2607-2618.1999 PMC85295

[14] Soini H, Pan X, Teeter L, Musser JM, Graviss EA. Transmission dynamics and molecular characterization of *Mycobacterium tuberculosis* isolates with low copy numbers of IS*6110.* J Clin Microbiol. 2001;39:217–21. 10.1128/JCM.39.1.217-221.2001 11136774 PMC87705

[15] Sola C, Horgen L, Goh KS, Rastogi N. Molecular fingerprinting of *Mycobacterium tuberculosis* on a Caribbean island with IS6110 and DRr probes. J Clin Microbiol. 1997;35:843–6. 9157139 10.1128/jcm.35.4.843-846.1997 PMC229687

[16] Beggs ML, Eisenach KD, Cave MD. Mapping of IS*6110* insertion sites in two epidemic strains of *Mycobacterium tuberculosis.* J Clin Microbiol. 2000;38:2923–8. 10921952 10.1128/jcm.38.8.2923-2928.2000 PMC87149

[17] Soini H, Pan X, Amin A, Graviss EA, Siddiqui A, Musser JM. Characterization of *Mycobacterium tuberculosis* isolates from patients in Houston, Texas, by spoligotyping. J Clin Microbiol. 2000;38:669–76. 10655365 10.1128/jcm.38.2.669-676.2000 PMC86172

[18] Soini H, Musser JM. Molecular diagnosis of mycobacteria. Clin Chem. 2001;47:809–14. 11325882

